# A roadmap for assessing the diagnostic usefulness of neurosensory testing and an updated method for exposure assessment among vibration-exposed workers in northern Sweden

**DOI:** 10.1080/22423982.2024.2403793

**Published:** 2024-09-12

**Authors:** Hans Pettersson, Ingrid Liljelind, Tohr Nilsson, Albin Stjernbrandt

**Affiliations:** Section of Sustainable Health, Department of Public Health and Clinical Medicine, Umeå University, Umeå, Sweden

**Keywords:** Health surveillance, neurological symptoms, screening, hand-arm vibration syndrome, measurements, Sweden, occupational health

## Abstract

Workers who use handheld vibrating machines such as grinders, hammers and chainsaws expose themselves to hand-arm vibration (HAV). Exposure to HAV may cause injuries to both the neurological and the vascular system. The occupational health services (OHS) in Sweden use a formal guideline for secondary prevention focusing on early detection of vibration-related injuries. The guide includes several screening tools, such as a screening questionnaire, clinical examinations, and laboratory tests. There are no studies, to our knowledge, on the diagnostic value of the separate items on symptoms in the screening questionnaire in relation to the laboratory tests or the clinical examinations performed during a medical examination among patients exposed to HAV. Furthermore, the recently presented ISO standard for HAV measurements (ISO/TR 18,750) has only been tested for vascular injuries and not neurological injuries. This research roadmap aims to evaluate separate items in a screening questionnaire on neurological symptoms in relation to laboratory and clinical tests among HAV exposed workers in the Arctic region of Northern Sweden. It also covers a comparison of the dose-response of the current ISO 5349–1 measurement standard and the new suggested standard ISO/TR 18,750 with the neurosensory outcomes. This manuscript describes the study rationale, design, methods, and significance.

## Introduction

Health surveillance is an important part of both primary and secondary preventions among workers in the Arctic and globally. Health surveillance includes screening of injuries and symptoms, using clinical testing or questionnaire items. Questionnaire items have the benefit of low costs and can also be less time-consuming than medical testing. This paper describes a research roadmap for evaluating questionnaire items on neurological symptoms with clinical examinations and laboratory neurosensory testing among vibration-exposed workers in the Arctic region of Northern Sweden. Furthermore, this paper also includes a rationale for evaluating neurosensory risk from exposure to hand-arm vibration (HAV) using current and a proposed measurement standard.

### Diagnostic value of questionnaire items, clinical examinations, and laboratory neurosensory testing among vibration-exposed workers

Workers who use handheld vibrating machines such as grinders, hammers, and chainsaws expose themselves to HAV. Such exposure is common in the construction, manufacturing and service industries (i.e. maintenance of roads or vehicles). Exposure to HAV occurs among the working age general population in the Arctic region of Northern Sweden in industries such as industry, agricultural, forestry, and fishery workers [1]. Miners at open-pit mines in Norway and Sweden are also commonly exposed to HAV [2]. A HAV dose with enough exposure magnitude and duration may cause both vascular and neurological as well as musculoskeletal symptoms and injuries to the hand and arm [3]. Vascular and neurological symptoms in the hands have been reported to be common in the working-age general population of the Arctic northern Sweden as well as in specific lines of works such as open-pit mining in the northern Norway and Sweden [1,2]. Few of the workers who contract injuries experience improvement over time [4]. Among males in the Swedish workforce, vibration injuries including carpal tunnel syndrome constitute 62% of all approved compensation claims for occupational disease [5].

The large number of workers exposed to hazardous vibration and the severity of the injuries from HAV, together with the unfavourable prognosis and the lack of medical treatment, have led to a mandatory directive 2002/44/EC on how to prevent vibration injuries [6]. In addition to the determination and assessment of vibration exposure, the EU directive states that the employer must perform a risk assessment on workers who may be at risk of injuries due to HAV. The risk assessment includes gathering information on exposure to HAV and other associated factors that increase the risk of injuries due to HAV, such as low temperatures and strenuous work postures. The risk assessment may conclude that the worker should be offered health surveillance. Health surveillance includes a medical examination intended to diagnose early signs of injury due to HAV and is performed by a medical doctor in cooperation with other occupational health services (OHS) professionals [7].

The medical examination consists of a patient's history, information from questionnaires, physical examination, and their current and earlier exposure to HAV. The examination aims to identify important factors for the worker’s wellbeing when using hand-held vibrating machines. The gathered information from the medical examination is a basis for evaluating whether the work constitutes a health risk or if it is to be advised for the worker to continue HAV exposure, and the examination also provides a basis for further preventive work measures. Because neurosensory injury is often considered permanent, the examination should also identify early signs of injury to prevent further deterioration [3,8].

Even with the implementation of the EU directive, the preventive measures have not been successful since the majority of compensated work-related injuries in Sweden are still related to HAV exposure [5].

The OHS in Sweden use guidelines for the early detection of vibration-related injuries, which include a screening questionnaire as well as a descriptive manual on how to conduct medical examinations. Such medical examinations are to be performed before exposure is begun and then every third year. However, every other recurrent examination can be substituted with an abbreviated health screening based on a screening questionnaire.

At present, it is still unclear if the items in the screening questionnaire and the medical examination are sufficient in detecting neurological symptoms among workers exposed to HAV. Earlier studies have focused on the screening for vascular symptoms with clinical tests [9] or validated screening questions for vascular and nerve damage symptoms against a physician’s assessment [10]. The physicians used a medical history questionnaire and standardised quantitative testings such as thermal aesthesiometry, measurement of vibrotactile thresholds and grip strength [10]. The research group around Ron House and Aaron Thompson have recent publications on factors effecting clinical testing of neurovascular symptoms [11]. To our knowledge, there is no previous study on the diagnostic predictive value of questionnaire items in screening tools for neurological symptoms and its relation to the laboratory tests or clinical examinations performed during a medical examination among patients exposed to HAV.

### New method for exposure assessment

The risk assessment in the EU directive [6] includes measurements of the exposure to HAV for workers using vibrating handheld tools. Measurements of HAV are conducted according to ISO 5349–1 [12]. Unfortunately, although neurosensory vibration injury appears to be more common, the risk-modelling appendix of the ISO-5349-1 standard only includes injuries in the form of vibration-induced white fingers, a form of secondary Raynaud’s phenomenon. A review on the exposure-response effect of vascular and neurosensory injuries from exposure to HAV emphasised the need for a new risk model that also includes neurosensory injuries [3]. The authors also concluded that the current action and limited values for HAV underestimate the risk of neurological injuries. One explanation might be the way exposure to vibration is measured. The presently suggested measurement standard, ISO/TR 18,750, includes a broader frequency spectrum for HAV exposure [13] compared to the ISO 5349 standard. Earlier studies comparing these measurement standards have been few and focused on vascular injuries in the form of Raynaud’s phenomenon and included mainly forestry and quarry workers [14–16].

Based on the lack of studies on the screening questionnaire for neurosensory symptoms from exposure to HAV and its relation to medical examinations and the lack of studies on exposure-response comparisons between the current ISO standard 5349–1 and the new suggested standard ISO/TR 18,750, a new project is established to answer these questions. This paper describes the rationale and methods for the study.

## Objectives

This research roadmap covers the process of evaluating separate items in a screening questionnaire on neurological symptoms in relation to laboratory and clinical tests among workers in the Arctic region of northern Sweden. It also covers a comparison of the dose-response of the current ISO 5349–1 measurement standard and the new suggested standard ISO/TR 18,750 with the neurosensory outcomes. This manuscript describes the study rationale, design, methods, and significance.

## Methods

### Implementation

**Figure 1.**
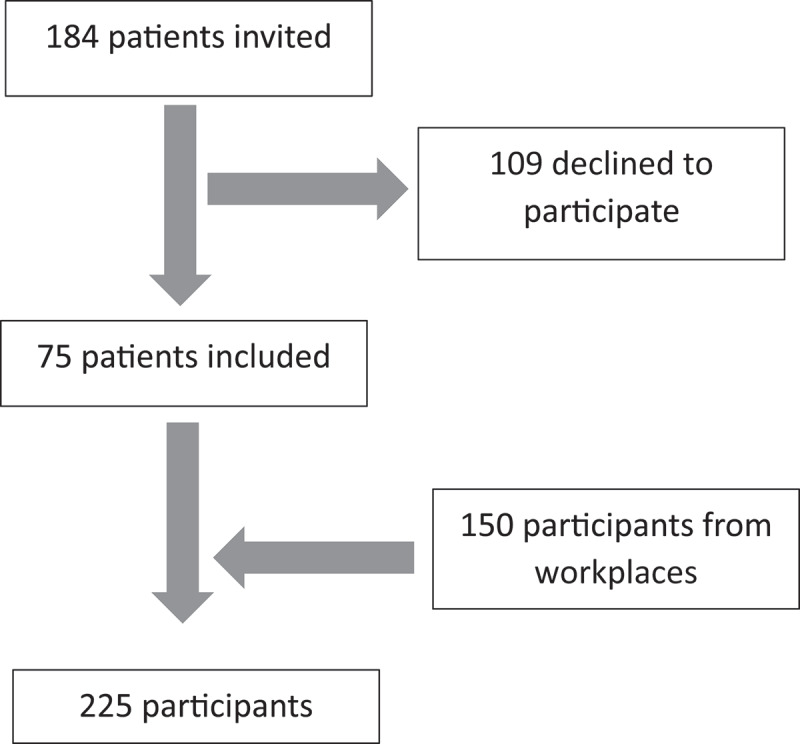
Flow chart on included participants.

The project was conducted in the Arctic region of the northern counties of Sweden, including Norrbotten, Västerbotten and also Västernorrland. The branches of industry and companies invited into the project had workers using different types of handheld vibrating tools as well as different durations of use. Invited companies included workers in the construction industry, car and tyre workshops, heavy manufacturing, and rental companies that supply handheld vibrating tools for rent. The invited workers from the companies had their medical examinations performed either at the Occupational and Environmental Health Clinic at the University Hospital of Umeå in the Arctic region of northern Sweden or at the site for the companies in Västernorrland from April 2022 to February 2024. A convenience sampling strategy was used at visited workplaces, which was not dependent on whether subjects had symptoms of neurosensory HAV injury or not. All patients at the clinic who had been referred because of suspected HAV injury were invited to the study from August 2021 until December 2023. A Flowchart on participants is presented in Figure 1. The subjects were assessed using clinical tests, quantitative sensory testing (QST) and laboratory tests that were related to the screening questionnaire neurological questions. Field measurements of HAV exposure were also conducted at companies. All participants were informed about the study before giving written consent to participate. The study protocol was approved by the Swedish Ethical Review Authority, Sweden (DNR 2021–01463).

Among the participants who were patients at the clinic, the clinical and laboratory tests were performed by the medical doctors employed at the clinic. All medical doctors were informed about the correct protocol when performing the clinical tests before the study began. QST was performed by four different staff members at the clinic with various years of experience with the test. All four staff members were instructed on how to perform testing correctly. For all participants invited from companies, the clinical tests were performed by two researchers who had been trained on how to perform the tests according to protocol. QST was performed by one researcher who was instructed and trained by an experienced staff member at the clinic.

### Questionnaires

All participants answered the screening questionnaire for neurosensory symptoms from exposure to HAV, a health questionnaire, and a questionnaire on handheld vibrating machines used by the participants during their working lives.

### Screening questionnaire for neurological symptoms from exposure to HAV

The screening questionnaire consisted of seven Likert-type questions of different neurological symptoms. Each question consisted of the following phrase: “Do you have any of the following?”. The seven statements were as follows: “a) Impaired ability to feel touch in fingers/hand?”, “b) Impaired ability to feel heat in fingers/hand?”, “c) Impaired ability to feel cold in fingers/hand?”, “d) Reduced strength in fingers/hand?”, “e) Pain when you get cold in fingers/hand?”, “f) Difficulty with fastening buttons?” and “g) Impaired ability to feel vibration in fingers/hand?”. Possible answers included “No”, “Insignificant”, “Somewhat” and “Quite a lot”. A positive response for symptoms was “Somewhat” or “Quite a lot” [17].

### Health questionnaire

The health questionnaire included questions on weight, height, tobacco use (including both smoking and oral moist snuff), previous diagnoses related to neurological symptoms (e.g. multiple sclerosis, nerve injury and carpal tunnel syndrome), thyroid dysfunction, diabetes mellitus, vitamin B deficiency and alcohol dependency) and treatments (e.g. cytostatics and immunosuppressive drugs). The participants were also asked in the questionnaire if they used any daily medication or if they had any first-degree relatives (mother, father, or siblings) who had a neurological disorder.

### Questionnaire on exposure to hand-arm vibration

In the exposure questionnaire, participants were asked to name the handheld vibrating machine types used and models of the machines and estimate the duration in minutes per day they operated the stated machines during different periods in their working life (i.e. the last week, month, year and lifetime).

### Study protocol

All clinical examinations were performed indoors with an ambient temperature of 20–22°C. The skin temperature of the participants’ right and left index fingers were measured with an infrared thermometer (Testo 845, Alton Hampshire, UK) and had to be at or above 28°C before examinations would start. If the skin temperature was below 28°C, the fingers would warm. All participants were informed beforehand to avoid nicotine, caffeine, and strenuous physical activity 2 hours before the clinical tests and to avoid using handheld vibrating machines during the day of the examinations.

### Clinical test

Participants were asked to sit comfortably in a chair with their hands on a cushion or bunk with their palms upwards and asked to look away or close their eyes during testing. Tests were performed on the right and left index and little fingers. A deviant value for each test was defined as the mean value ± 2 standard deviations.

The participants’ perception of touch was examined with a five-piece set of Semmes-Weinstein Monofilaments (Touch-Test®, North Coast Medical, Gilroy, USA) and two-point-discrimination (Touch-Test® Two-Point Discriminator) on the palm side of the fingertips. The monofilament was pressed down until it bent slightly for 1–1.5 seconds on the skin between the vortex of the fingerprint and the tip of each finger. Five target forces were used: 0.07, 0.4, 2.0, 4.0 and 300 grams. The participants were asked to tell the examiner if they sensed the monofilament. If they could not sense any of the three attempts, the examiner changed the monofilament to one with an increased force. An abnormal result was defined as the inability to perceive 0.4 grams of target force. Two-point discrimination was tested by randomly applying one or two spikes, starting with a 5 mm separation. The pressure applied was just enough to cause slight blanching of the skin. If the participant could discriminate a certain two-point distance two times, the examiner tested a lesser distance. If the participant could not discern if it was one or two spikes, the examiner increased the distance. The spikes were fixed on a sturdy plastic disc, with distances between spikes ranging from 2 up to 8 mm. The spikes had a diameter of around 0.5 mm. The ability to discriminate between warmth (40°C) and cold (25°C) was tested with temperature rollers (Rolltemp®, Somedic SenseLab AB, Sösdala, Sweden) on the palmar side. The examiner applied either the warm or cold roller for 1 second at the vortex of the fingertip and asked the participant if they sensed a warm or cold sensation. If the participant could not discriminate between warm and cold rollers three times, the examiner continued the test on the middle phalange, proximal phalange, palm, and wrist. The ability to sense vibrations was tested using a 64 Hz tuning fork (Rydel-Seiffer). The tip of the fork was applied perpendicular to the fingertip and held until the participant told the examiner that they could not sense any vibration. This tuning fork has a nine-degree scale and when vibrating, a triangle is visible along the scale. When the participant tells the examiner that they cannot feel any vibration, the examiner notes what number the triangle is indicating. The test was done three times for each finger.

Hand grip strength was tested using a hydraulic dynamometer (Jamar®, Sammons Preston, Rolyan®, Bolingbrook, IL), with the participant sitting straight in a chair with perpendicular elbows, informed to grip as hard as they could three times with 10 seconds rest between each grip strength test. The test was first performed on the dominant hand and then on the other hand.

### Quantitative sensory testing

QST is a semi-objective method to measure the participants’ thermal and vibration perception thresholds. The measurements were performed on the palmar side on the right and left index and on the little fingers.

To measure the vibration perception thresholds (VPT), each participant was instructed to lay the vortex of the fingerprint on a vibrating probe (VibroSense Meter MATLAB®, VibroSense Dynamics AB, Malmö, Sweden) and rest their elbow on a support. Seven different frequencies, ranging from 8 to 500 Hz, were tested using the von Békésy method in compliance with ISO 13,091–1 (2001). A Z-score for each frequency as well as an average Z-score for all frequencies were calculated.

Warm detection thresholds (WDT) and cold detection thresholds (CDT) were determined on the distal and palmar parts of the fingers using a 25 × 50 mm thermode attached to the SenseLab Modular Sensory Analyzer (SENSELab MATLAB®, Somedic SenseLab AB, Sösdala, Sweden). Participants were instructed to press a button when they sensed a change in temperature. The method-of-limits was used, where the first five stimuli had a temperature change towards colder and the next five stimuli had a temperature change towards warmer. The temperature stimuli changed by 1°C per second. The starting temperature was 32 ± 0.1°C with a range of 10−50°C. After each press of the button, the temperature returned to the starting temperature and the stimuli started at random time intervals between 4 and 6 seconds.

### Laboratory tests

The cold pain provocation test (CPPT) studies the reaction to cold by measuring the pain threshold and pain tolerance. Contraindications were ischaemic or congestive heart disease and implanted cardiac devices. If a participant’s blood pressure was above 180/110 mmHg or under 100/50 mmHg, a medical doctor was consulted. The participant sat next to a circulating cooling water bath with a temperature of 3 ± 0.1°C (Alpha RA 12, Lauda, Germany) and was instructed to immerse the right hand with water up to their wrist level and not move their hand or touch the sides or bottom of the water bath. The verbal instruction was as follows: “If you feel pain or discomfort that makes it unbearable to keep your hand in the water, you can take it out”. With the left hand, the participant was instructed to rate the pain in the immersed hand using a 100 mm digital visual analogue scale (VAS) ranging from “no pain” to “worst pain imaginable” during the immersion of the hand. The maximum immersion time was 60 seconds, and the examiner measured the immersion time and VAS ratings.

A Purdue pegboard (Model 32020A) was used for testing manual dexterity. The board was placed 5 cm from the end of the table next to the participant. The participant was instructed to place as many pins as possible for 30 seconds for each hand and then use both hands. The test began with the dominant hand. When using both hands, the participant was asked to place two pins simultaneously with both hands.

### Exposure assessment

The reported tools and, if possible, also models specified by the participants in the exposure questionnaire were measured with a six-channel human vibration metre (Svantek 106D, Warsaw, Poland) and analysed in MATLAB® (R2022b, The MathWorks Inc., USA) with an applied frequency weighting according to ISO 5349–1 [12] as well as ISO/TR 18,750 [13]. The measurement was performed while the tools were used by the operator when performing different work tasks. If it was not possible to measure the same model as stated by the participant, the measurement was performed on a machine of similar type or used earlier measurements from databases or standards. The measured vibration level using the frequency weighting from both standards was compared by machine type. The daily equivalent exposure level, A [8], for each period in the exposure questionnaire, was calculated according to the international standard 5349–1 [12] and according to the proposed standard ISO/TR 18,750 [13].

### Analyses

*Screening questionnaire for neurosensory symptoms and its relation to clinical tests*

For each question in the screening questionnaire, the sensitivity, specificity, positive and negative predictive values, and positive and negative likelihood ratios were calculated by comparing the respective results of the clinical, laboratory and QST tests as a basis for ruling in or ruling out the diagnosis of neurosensory injury due to HAV exposure (Table 1).

**Table 1.** Planned evaluation of diagnostic performance for each type of diagnostic tool.

Screening questionnaire	Clinical tests	Quantitative sensory testing or laboratory tests
a) Impaired ability to feel touch in fingers/hand?	Monofilament	
b) Impaired ability to discriminate heat in fingers/hand?	Warm roller	Warm detection threshold
c) Impaired ability to discriminate cold in fingers/hand?	Cold roller	Cold detection threshold
d) Reduced strength in fingers/hand?	Grip strength	
e) Pain when cold in fingers/hand?		Cold pain provocation test
f) Difficulty with fastening buttons?	Two-point discrimination	Purdue pegboard
g) Impaired ability to discriminate vibration in fingers/hand?	Tuning fork	Vibration perception thresholds

### Dose-response

This project will study which of the measurement standards, the current (ISO 5349–1) or the proposed standard (ISO/TR 18,750:2017), provides the best risk assessment model for neurosensory injury due to HAV exposure [12,13]. The outcome will be the seven screening questions covering impaired discrimination of touch, temperature, vibration, grip strength, dexterity, and cold-induced pain. The exposure for different periods of each participant will be calculated as the daily equivalent 8-hour exposure, A [8], based on the exposure questionnaire. The A [8] values will be calculated from the estimated time and vibration levels of each machine type stated by the participant [12,13]. The vibration exposure levels will also be quantified using the frequency weighting as stated in the current and proposed standards [12,13].

## Results

Among subjects visiting the Occupational and Environmental Health Clinic in the Arctic region of northern Sweden due to suspected neurosensory HAV injury, 184 were asked to participate and 75 provided written informed consent, giving a participation rate of 41%. At workplaces, a convenience sampling strategy was used that was not dependent on whether subjects had symptoms of neurosensory HAV injury or not. In this manner, additional 150 subjects were recruited. Descriptive data are presented in Table 2.

**Table 2.** Descriptive data on participants.

		Participants from the clinic	Participants from workplaces	All participants
		*N (%)*	*N (%)*	*N (%)*
Number of participants		75 (33)	150 (67)	225 (100)
Gender (male/female)	Male	67 (89)	141 (94)	208 (92)
	Female	8 (11)	9 (6)	17 (8)
Occupational group	Carpenters	33 (44)	99 (66)	132 (59)
	Mechanics	18 (24)	16 (11)	34 (15)
	Welders	7 (9)	35 (23)	42 (19)
	Other	17 (23)	0 (0)	17 (8)
Age group (years)	18–30	9 (12)	59 (39)	68 (30)
	31–40	17 (23)	41 (27)	58 (26)
	41–50	14 (19)	13 (9)	27 (12)
	51–60	27 (36)	27 (18)	54 (24)
	61-	8 (11)	10 (7)	18 (8)
*Daily exposure last year (m/s2)		2.7 (2.5)^†^	3.1 (2.1)^††^	3.0 (2.2) ^†††^

*Mean and std. ^†^2 missing values. ^††^7 missing values. ^†††^9 missing values.

## Significance of the project

Health surveillance is an integral part of preventing occupational injuries due to HAV exposure. The Arctic area covers a wide range of industries such as infrastructure, maintenance construction, manufacturing and mining where many workers are exposed to HAV and therefore at risk of neurological symptoms and injuries. Most earlier studies on screening of HAV-related injuries and symptoms have not covered neurological symptoms or work populations in the Arctic region.

The result from this project can be used for workers’ protection in the Arctic region of northern Sweden since the project includes workers from occupations such as construction, manufacturing and mechanics, all common industries in this setting [1].

The current project was developed to study the relationship between the screening questionnaire for neurosensory injuries and different kinds of medical examinations among workers in the Arctic northern Sweden. This project includes 75 participants who had been referred to the clinic in Umeå because of suspected injuries related to HAV exposure. Another 150 participants were recruited from different companies. To include participants with and without neurological symptoms in this study was important since the analyses required a variety of different outcomes from the different questions in the screening questionnaire as well as medical examinations. Most of the participants were under 40 years old but many still showed signs of neurosensory injury, which is alarming.

Most participants worked as carpenters but there were also many mechanics and welders included. These three groups were also the most common among participants from the clinic. Different occupations use different machine types which is important for the analysis of the relation between neurological symptoms and HAV exposure calculated by the current and the proposed measurement standard. Different machine types have different frequency spectrums that will affect the calculation of the HAV exposure levels when using the current and proposed measurement standards. This variety of machine types will improve the analysis of the relationship between neurological symptoms and HAV exposure since we included many different machines from different occupations.

The daily exposure levels last year suggested that many participants had hazardous HAV exposure at work, often exceeding the action value. This emphasises the importance of preventive measures such as detecting early symptoms of neurological injury and improving the understanding of exposure–response relations.

The screening questionnaire and medical examination, alone or together, are important preventive tools to find early signs of neurosensory injuries among workers exposed to HAV. There are no previous studies on the relationship between the screening questionnaire and corresponding clinical examinations. We hope that this project will contribute to the understanding of the quality of the screening questionnaire in relation to the clinical tests, QST used by the OHS in Sweden and laboratory tests that are not in clinical use today. We also hope to be able to suggest possible improvements in the screening methods. Thus, this project may increase the quality of screening among HAV-exposed workers and offer a greater understanding of how exposure to HAV causes neurosensory injury. Since early detection of injury is paramount, we believe that the development of valid and sensitive diagnostic tools is an urgent matter.

This project will also compare the relationship of both current and proposed measurement standards of HAV with neurosensory outcomes. Earlier research of proposed standard ISO/TR 18,750 has been focused on the relation to vascular injuries from HAV, but this project is the first to study their relation to neurosensory outcomes.

The current project will increase our understanding of the relationship between the screening questionnaire for neurosensory injuries and different kinds of medical examinations among workers in the Arctic region of Northern Sweden. It will offer insight into whether the screening questionnaire can replace clinical testing to some extent. Future research may focus on improving both the screening questionnaire and the medical examinations for workers exposed to HAV in the Arctic northern Sweden and other regions. The current project will also cover modelling HAV levels with different methods and provide information that could be used to improve the current measurement standards.

## Data Availability

The collected data used during the current study can be made available upon reasonable request from the corresponding author.
